# Mitophagy in Parkinson’s Disease: From Pathogenesis to Treatment

**DOI:** 10.3390/cells8070712

**Published:** 2019-07-12

**Authors:** Jia Liu, Weijin Liu, Ruolin Li, Hui Yang

**Affiliations:** 1 Department of Neurobiology School of Basic Medical Sciences, Beijing Institute for Brain Disorders, Capital Medical University, Beijing 100069, China; 2 Center of Parkinson’s Disease Beijing Key Laboratory of Neural Regeneration and Repair, Beijing Key Laboratory on Parkinson’s Disease, Key Laboratory for Neurodegenerative Disease of the Ministry of Education, Beijing 100069, China

**Keywords:** mitophagy, Parkin, Parkinson’s disease, PINK1, treatment

## Abstract

Parkinson’s disease (PD) is the second most common neurodegenerative disease. The pathogenesis of PD is complicated and remains obscure, but growing evidence suggests the involvement of mitochondrial and lysosomal dysfunction. Mitophagy, the process of removing damaged mitochondria, is compromised in PD patients and models, and was found to be associated with accelerated neurodegeneration. Several PD-related proteins are known to participate in the regulation of mitophagy, including PINK1 and Parkin. In addition, mutations in several PD-related genes are known to cause mitochondrial defects and neurotoxicity by disturbing mitophagy, indicating that mitophagy is a critical component of PD pathogenesis. Therefore, it is crucial to understand how these genes are involved in mitochondrial quality control or mitophagy regulation in the study of PD pathogenesis and the development of novel treatment strategies. In this review, we will discuss the critical roles of mitophagy in PD pathogenesis, highlighting the potential therapeutic implications of mitophagy regulation.

## 1. Introduction

Parkinson’s disease (PD) is one of the most common neurodegenerative disorders in the world, and is characterized by typical motor deficits, including bradykinesia, tremors, rigidity, and postural instability, and a series of non-motor symptoms such as dysosmia, constipation, and depression. The pathological features of PD include the progressive loss of dopaminergic neurons in the substantia nigrapars compacta (SNpc) and the formation of Lewy bodies [1].

PD can be subdivided into familial and sporadic PD. Epidemiological studies have shown that about 10% of PD cases are inherited, whereas the remaining cases are sporadic. Although the etiology of PD remains unknown, it is believed to involve both genetic and environmental factors, and is associated with aging [2,3]. Thus far, several proteins have been identified as contributing to PD pathogenesis, including α-synuclein (α-syn), Parkin, PTEN-induced putative kinase (PINK)1, DJ-1, Leucine-rich repeat kinase (LRRK) 2, and others (Table 1). Many of these are known to participate in mitochondrial quality control or lysosomal functions [4]. Furthermore, familial PD patients with gene mutations usually show mitochondrial defects and impairment of the autophagic pathway [5,6,7,8], indicating that these two elements are critical components of PD pathogenesis.

## 2. Mitophagy Pathways

Mitophagy, the selective degradation of mitochondria via autophagy, is a key process for maintaining mitochondrial homeostasis. Mitochondrial turnover via this mechanism is regarded as a significant mechanism for maintaining neuronal health [9]. However, abnormal mitophagy accompanies neurodegeneration. [10] A growing number of studies have shown that autophagy dysfunction impairs mitochondrial homeostasis, and in turn, mitochondrial defects also impact lysosomal functions, suggesting a complex relationship between these processes. [11] Mitophagy impairment results in the progressive accumulation of defective mitochondria, leading to neuronal death and eventual neurodegeneration. In this review, we discuss the critical roles of mitophagy in PD pathogenesis, highlighting the potential therapeutic implications of mitophagy regulation.

**Figure 1:**
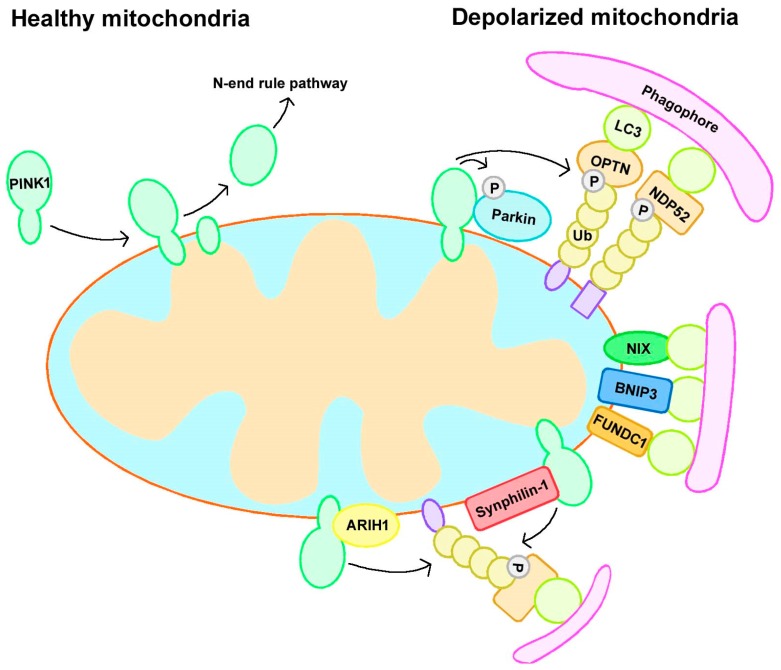
Mitophagy pathways. Mitophagy can be divided into Parkin-dependent or independent pathways. Under normal conditions, PINK1 localizes to mitochondria and is translocated to the mitochondrial inner membrane (MIM), where it is cleaved and subsequently degraded by an N-end rule pathway. However, when mitochondria become depolarized, PINK1 accumulates at the outer mitochondrial membrane (OMM) and recruits Parkin. Activated Parkin leads to the ubiquitination of substrates and the recruitment of autophagy receptors to initiate mitophagy. In addition, Parkin-independent mitophagy includes receptor-mediated and ubiquitin ligase-mediated mitophagy. BNIP3, BCL2/adenovirus E1B 19 kDa interacting protein 3; FUNDC1, FUN14 domain-containing protein 1; NDP52, nuclear dot protein 52 kDa; NIX, BCL2/adenovirus E1B 19 kDa interacting protein 3 like; OPTN, optineurin; Ub, ubiquitin.

Cells possess several mitophagy mechanisms, and different stresses promote mitophagy in distinct cellular contexts. Indeed, mitophagy can be divided into Parkin-dependent or independent pathways, with some crosstalk between them (Figure 1) [12,13].

### 2.1. Parkin-Dependent Mitophagy

In 2008, the Youle lab first identified the relationship of the PD-related gene Parkin and mitophagy, which was regarded as a landmark study in mitophagy [14]. Subsequently, several studies showed that another PD-related gene, PINK1, also participated in this process [15,16,17]. PINK1 is a serine/threonine kinase encoded by the *PARK6* gene. Parkin is an E3 ubiquitin ligase encoded by the *PARK2* gene. Mutations in PINK1 and Parkin result in autosomal recessive PD [18,19]. Loss of function of PINK1 or Parkin causes prominent mitochondrial pathology and loss of dopaminergic neurons [20,21,22], and subsequent studies discovered a crucial role for PINK1 and Parkin in mitophagy. In the current decade, much research related to PINK1/Parkin-mediated mitophagy has emerged, and this pathway is regarded as the most common and important pathway in mitophagy [23].

The PINK1/Parkin pathway regulates Ub-dependent mitophagy. The assembly of ubiquitin chains on mitochondria is important for the removal of damaged mitochondria via this pathway. This assembly requires three significant elements: PINK1 as a mitochondrial damage sensor, Parkin as a signal amplifier, and ubiquitin chains as the signal effector [24]. PINK1 has a mitochondrial targeting sequence that can guide it to mitochondria, and it triggers mitophagy by sensing mitochondrial depolarization or the accumulation of reactive oxygen species (ROS). Under normal conditions, PINK1 localizes to mitochondria and is translocated to the mitochondrial inner membrane (MIM), where it is cleaved and inactivated by the MIM protease presenilin-associated rhomboid-like protein (PARL) and subsequently degraded by an N-end rule pathway [25,26]. However, when mitochondria become depolarized, PINK1 cannot be translocated to the MIM and cleaved, resulting in its accumulation at the outer mitochondrial membrane (OMM) and the subsequent recruitment and phosphorylation of Parkin [16], which is localized in the cytoplasm and in an inactivated form in healthy mitochondria [27]. Activated Parkin subsequently leads to the ubiquitination of mitochondrial membrane proteins and the recruitment of autophagy receptors such as optineurin (OPTN) and nuclear dot protein 52 kDa (NDP52) to mitochondria [28,29], followed by the formation of LC3-positive phagophores that degrade mitochondria via the lysosome [14,24].

### 2.2. Parkin-Independent Mitophagy

Although Parkin is considered to be a crucial regulator of mitophagy, growing evidence shows that mitophagy can occur in the absence of Parkin, which is known as Parkin-independent mitophagy [13]. Generally, Parkin-independent mitophagy includes receptor-mediated and ubiquitin ligase-mediated mitophagy.

#### 2.2.1. Receptor-Mediated Mitophagy

Several protein receptors have a LIR (LC3-interacting region) motif, which allows them to bind to LC3 to induce mitophagy [30]. B-cell lymphoma 2 (BCL2)/adenovirus E1B 19 kDa interacting protein 3 (BNIP3) and its homolog BCL2/adenovirus E1B 19 kDa interacting protein 3 like (NIX/BNIP3L) are BH3-only proteins belonging to the B-cell lymphoma 2 (BCL-2) family, and both of them can induce mitophagy in HeLa cells lacking Parkin expression. They can insert into the OMM via their C-terminus, and their N-terminus has an LIR domain to facilitate binding to LC3 or GABARAP [31,32]. FUN14 domain-containing protein 1 (FUNDC1) is a mitophagy receptor that responds to hypoxia-induced mitophagy; it can localize to the OMM and has an LIR domain allowing its association with LC3 [33]. Autophagy and Beclin 1 regulator 1 (AMBRA1), an upstream autophagy regulator, can interact with LC3 through an LIR motif; interestingly, AMBRA1 can trigger both Parkin-dependent and Parkin-independent mitophagy [34,35]. In addition to these receptors, some lipids can also bind to LC3 to induce mitophagy, such as cardiolipin. Under normal conditions, cardiolipin is localized in the IMM, and it is exported to the OMM upon mitochondrial stress and binds to LC3 to trigger mitophagy directly [36].

#### 2.2.2. Ubiquitin Ligase-Mediated Mitophagy

An earlier study suggested that Parkin is indispensable for mitophagy induction, but in 2015, researchers found that PINK1 can recruit NDP52 and optineurin to mitochondria to trigger mitophagy directly, independent of Parkin; these data indicate that Parkin is not indispensable for mitophagy, but rather acts to amplify this signal [37]. In addition to Parkin, there are several other ubiquitin E3 ligases that function in mitophagy. A novel E3 ligase called ARIH1 was found to participate in mitophagy in a PINK1-dependent manner [38]. Mitochondrial ubiquitin ligase activator of NF-kB1 (MUL1) is another ubiquitin E3 ligase on the OMM, and it can compensate for the loss of Parkin/PINK1 loss in a PD model to rescue their mutation-induced phenotypes [39]. Synphilin-1 can interact with PINK1 and be recruited to the mitochondria to promote PINK1-dependent mitophagy. This mitophagy pathway was independent of PINK1-mediated phosphorylation and Parkin [40].

## 3. Mitophagy in PD Pathogenesis

The mitochondrion is the critical organelle for generating energy for cellular processes, and mitochondrial functionality determines whether a cell survives or dies. Disturbances to mitochondrial homeostasis are known to contribute to several neurodegenerative diseases, including PD [41,42]. Mitochondrial respiratory chain deficits, especially reductions in the activity of complex I, were found in post-mortem brains from sporadic PD patients [43], indicating a significant role of mitochondria in PD pathogenesis. In addition, one of the earliest studies observed mitochondria within autophagosomes in the neurons of PD patients, indicating a potential link between autophagy, damaged mitochondria, and PD pathogenesis [44]. Subsequently, abnormal mitophagy was observed in several PD models, including environmental or genetic forms [36,45,46,47,48,49,50].

**Figure 2:**
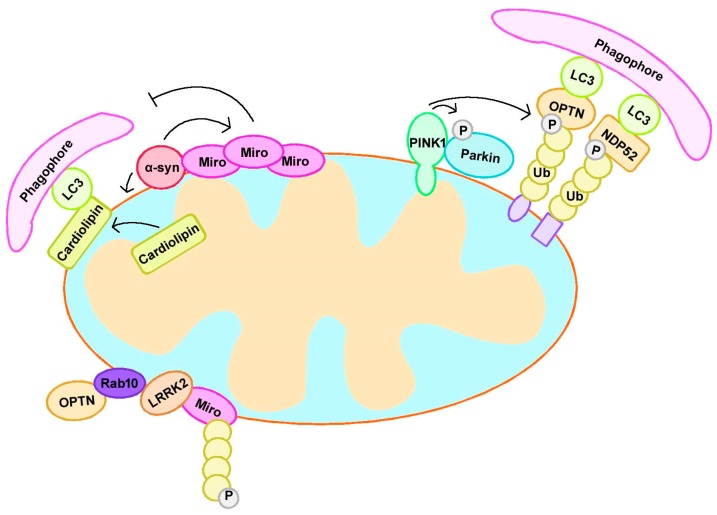
PD-related proteins participate in mitophagy. PINK1 accumulates at the outer mitochondrial membrane (OMM) and recruits Parkin to initiate mitophagy. α-syn interacts with Miro and upregulates Miro protein levels, leading to excessive, abnormal Miro accumulation on the mitochondrial surface and delayed mitophagy. Mitochondrial localized α-syn also promotes cardiolipin exposure on OMM; the latter further recruited LC3 to mitochondria and induced mitophagy. LRRK2 interacts with RAB10, which binds OPTN to induce mitophagy. LRRK2 interacted with Miro and contributed to its removal via mitophagy. NDP52, nuclear dot protein 52 kDa; OPTN, optineurin; Ub, ubiquitin.

Most PD-associated gene mutations participate in mitochondrial dysfunction and mitophagy disorder, including PINK1 and Parkin [51] (Table 2). Therefore, a thorough understanding of how these genes participate in mitochondria quality control or mitophagy modulation is critical in the study of PD pathogenesis and for developing new treatment strategies (Figure 2).

### 3.1. PINK1 and Parkin

As mentioned above, PINK1 and Parkin play critical roles in the process of mitophagy. In the following, we will discuss the canonical mitophagy regulation and non-canonical mitophagy regulation mediated by PINK1 and/or Parkin.

#### 3.1.1. Canonical Mitophagy Regulation

PINK1-dependent activation of Parkin is the major pathway leading to mitophagy, as described above. PINK1 plays a dual role of phosphorylating Ub and Parkin on damaged mitochondria. PINK1 phosphorylates Ub or poly-Ub chains at serine 65 (Ser65), and Parkin mediates a feed-forward mechanism to produce poly-Ub chains, thereby amplifying mitophagy signals [52]. Therefore, PINK1 and Parkin cooperatively recognize damaged mitochondria with phosphorylated poly-Ub (p-Ub) chains under stress. Studies on human post-mortem brain specimens have shown that distinct pools of p-Ub-positive structures co-localized with markers of mitochondria, autophagy, and lysosomes. Furthermore, p-Ub structures accumulated in the brains of Lewy body disease patients in an age-dependent and Braak stage-dependent manner, suggesting that p-Ub may be a biomarker for mitochondrial impairment in aging and disease [53]. A recent study showed that a novel mutant of PINK1, I368N, cannot be stabilized on the OMM upon mitochondrial stress due to conformational changes in its active site that fail to allow for polyubiquitination [54]. The RING1-IBR (in-between-RING) domain of Parkin preferentially binds to ubiquitin in a phosphorylation-dependent manner [55]. Another report showed that p-Ub binds to the RING1 of Parkin at His302 and Arg305, and promotes the disengagement of the UBL from RING1 and Parkin phosphorylation [56].

Ubiquitination is a reversible process, and several deubiquitinating enzymes (DUBs) are known to act in mitophagy. In addition to the roles of PINK1 and Parkin, the formation of p-Ub or poly-Ub chains is balanced by the activities of deubiquitinases. DUBs such as USP30 and USP35 oppose mitophagy by eliminating Ub chains generated by Parkin on OMM [57,58]. Parkin interacts with USP8 and removes K6-linked ubiquitin chains to regulate its own activity [59,60]. USP15 is another antagonist of Parkin; it does not affect the ubiquitination or translocation of Parkin, but rather inhibits Parkin-mediated mitochondrial ubiquitination [61]. A recent study showed that PTEN-long (PTEN-L), a novel PTEN isoform, regulated negatively mitophagy by dephosphorylating p-Ub via its protein phosphatase activity. PTEN-L prevented Parkin mitochondrial translocation, allevieated Parkin phosphorylation, inactivated Parkin activity, and further disrupted the feedforward of mitophagy [62,63]. Therefore, the balance between ubiquitination and deubiquitination regulates mitophagy and mitochondrial homeostasis.

Parkin has an equivalent Ser65 residue that is similar to ubiquitin, and is located within its N-terminal ubiquitin-like (UBL) domain; this residue is phosphorylated by PINK1, resulting in an open and active conformation [27,64]. Three substitutions in the UBL domain of Parkin (G12R, R33Q, and R42P) were found to significantly decrease PINK1 ability to phosphorylate Parkin. Two other UBL domain substitutions (G12R and T55I) increased the autoubiquitination of Parkin, suggesting that these substitutions increase Parkin degradation [65]. Parkin S65N, a PD-associated mutation, cannot be activated by PINK1. In addition, mice overexpressing Parkin S65A, which is a mutant that cannot be phosphorylated by PINK1, exhibit selective motor deficits, highlighting the critical role of Parkin Ser65 phosphorylation in PD pathogenesis [66]. Furthermore, growing evidence has shown that PINK1–Parkin signaling is prominent in dopaminergic neurons compared with other neurons, suggesting the vulnerability of dopaminergic neurons to mitochondrial stress [67].

The activity of PINK1 can be regulated by its post-translational modification. PINK1 can phosphorylate itself to regulate its kinase activity; Ser228 and Ser402 sites can be autophosphorylated on truncated PINK1, but not on full-length PINK1, and these phosphorylated PINK1 residues further regulate the phosphorylation of Parkin and Ub, and are involved in the induction of mitophagy [68]. Mitochondrial dysfunction arises from increased levels of S-nitrosylated PINK1 (SNO-PINK1), which is a specific post-translational modification on PINK1 that inhibits its kinase activity. SNO-PINK1 formation was found to disrupt mitophagy by decreasing Parkin translocation to mitochondria, further contributing to neuron death [69].

Despite post-translational modification, PINK1 and Parkin are also modulated by other factors. Several studies demonstrated the interaction between p53 and parkin [70,71,72]; another study showed that PINK1 can be down-regulated by p53 directly via its transcriptional activity, and further modulated mitophagy [73]. Interestingly, one recent study for the first demonstrated that Parkin could also act upstream to PINK1 via its transcription factor function by the activation of presenilins promoters. It is regarded as a novel feedback loop between Parkin and PINK1 in the control of mitophagy [74].

#### 3.1.2. Non-Canonical Mitophagy Regulation

In addition to their known roles in mitophagy, Parkin and PINK1 may modulate mitophagy in other ways. The autophagy protein Beclin1 can interact with Parkin in the cytosol, and is involved in Parkin translocation to mitochondria [75]. Similarly, PINK1 also interacts with Beclin1 [76] at the mitochondria-associated membrane (MAM), which is a specific region between the ER and mitochondria involved in mitochondrial quality control, suggesting a novel role for PINK1 in mitophagy regulation [77]. In addition, a recent study showed that dopaminergic neurons and microglia exhibit a high degree of mitophagy, while basal mammalian mitophagy occurs independently of PINK1, suggesting the presence of other, yet-to-be-discovered mitophagy pathways [78].

#### 3.1.3. PINK1/Parkin-Mediated Mitophagy and Mitochondrial Dynamics

Mitophagy mediated by PINK1 and Parkin also involves changes in mitochondrial dynamics, which play a significant role in maintaining mitochondrial homeostasis, especially in neurons. Miro is an adaptor located on the outer mitochondrial membrane; it mediates mitochondrial motility under normal conditions, and is removed from damaged mitochondria upon stress to facilitate mitochondrial clearance via mitophagy. Miro turnover on damaged mitochondria is altered in PD patient-derived fibroblasts with Parkin mutations. Mitochondrial dysfunction triggers the Lys27-type ubiquitination of Miro on the OMM in a PINK1-dependent and Parkin-dependent manner. Additionally, Miro can stabilize phosphomutant versions of Parkin on the OMM, suggesting its role as a member of the Parkin receptor complex [79]. PINK1 was found to phosphorylate Miro, and phosphorylated Miro activated the proteasomal degradation of Miro in a Parkin-dependent manner [80]. Interestingly, the PINK1/Parkin pathway can quarantine damaged mitochondria prior to their clearance by preventing mitochondrial movement [81].

Fission and fusion control mitochondrial morphology, and their balance is critical for the mitochondrial network. Growing evidence suggests that PINK1 and Parkin are critical for modulating mitochondrial fission and fusion. Indeed, the mitochondrial fusion proteins mitofusins (MFN) are substrates of PINK1 and Parkin [82]. Parkin can ubiquitinate MFN1/2, and these polyubiquitin chains are phosphorylated by PINK1 in return [83]. The loss of PINK1 and Parkin causes increases in MFN abundance and damaged mitophagy processes in Drosophila [84]. PINK1 and Parkin mutations in PD patient-derived fibroblasts also impaired MFN1/2 ubiquitination [85]. Subsequently, these signals recruited autophagy receptors to mitochondria, such as P62 and optineurin (OPTN), leading to eventual degradation by lysosomes [86]. Parkin also interacts with and ubiquitinates dynamin-related protein 1 (Drp1), which is a critical fission-related protein, to promote its proteasome-dependent degradation independent of mitophagy [87].

MFN2 also functions as an endoplasmic reticulum (ER)–OMM tether, and its phosphorylation and ubiquitination can trigger the disassembly of MFN2 complexes from the OMM to dissociate mitochondria from the ER; these findings raise the possibility of a regulatory mechanism of mitochondria–ER contact related to PINK1/Parkin that is independent of mitophagy [88]. ER and mitochondria are more closely associated in primary fibroblasts from PD patients with Parkin mutations and Parkin knockout (KO) mice compared with controls. Moreover, the abundance of MFN2 in the MAM was found to be elevated in PARK2 KO tissue and was accompanied by increased Ca^2+^ transfer from the ER to mitochondria, suggesting that Parkin is directly involved in regulating ER–mitochondria contacts [89].

### 3.2. α-Synuclein

α-Synuclein (α-syn) is the main component of Lewy bodies, and its mutation, duplication, or triplication results in autosomal-dominant PD [90]. α-Syn accumulation contributes to dopaminergic neuron death; although the underlying mechanism remains obscure, growing evidence suggests that mitochondrial dysfunction plays a significant role [41]. Many studies have found that α-syn could translocate to mitochondria via its N-terminus, and impaired mitochondrial function [91,92]. Moreover, α-syn was also found to impair autophagy, particularly mitophagy, and the latter process was further exacerbated by this detrimental mechanism through the impaired removal of dysfunctional mitochondria [93,94].

α-Syn can impair mitophagy in numerous ways. In the neurons of PD patients, α-syn interacts with Miro via its N-terminus and upregulates Miro protein levels, leading to excessive, abnormal Miro accumulation on the mitochondrial surface and delayed mitophagy, suggesting that Miro is a target of α-syn-associated mitochondrial injury [95]. Overexpression of the A53T α-syn mutant results in p38 MAPK activation, and this mutant directly phosphorylated Parkin at serine 131 to disturb its function and mitophagy [96]. In A53T α-syn-overexpressing mice, α-syn accumulates on mitochondria to cause increased mitophagy and neuronal death, while these mitochondrial deficits can be rescued by silencing Parkin and overexpressing Mfn2 or a dominant-negative variant of Drp1 [46,97]. In A53T and E46K α-syn transgenic mice, α-syn accumulates on the mitochondrial membrane and promotes cardiolipin exposure on the mitochondrial surface. Cardiolipin exposure recruited LC3 to mitochondria and induced mitophagy [98]. Yeast overexpressing both the human wild-type SNCA gene and A53T mutant showed enhanced mitophagy activities [99]. These studies indicate the role of abnormal mitophagy in α-syn-mediated toxicity.

### 3.3. LRRK2

Mutations in LRRK2, which is a member of the leucine-rich kinase family, are a cause of autosomal dominant PD [100]. Growing evidence suggests that mutations in LRRK2 result in abnormal mitophagy, although the mechanism remains controversial. One study showed that the levels of autophagy markers p62 and LC3 were increased in induced pluripotent stem cell-derived dopaminergic neurons from PD patients with the G2019S mutation in LRRK2, which is the most common LRRK2 mutation related to PD, suggesting the involvement of abnormal autophagy in G2019S-induced neurotoxicity [101]. Another study showed that the number of fragmented mitochondria increased and mitophagic clearance was reduced in human neuroepithelial stem cells from PD patients carrying the G2019S mutation [102]. However, another study suggested that G2019S LRRK2 mutations increase mitophagy due to histone deacetylase activation [103]. In addition, a study of human iPSC-derived neurons showed that LRRK2 interacted with Miro and contributed to its removal. The G2019S mutation delayed mitophagy initiation, while the knockdown of Miro rescued the injury caused by G2019S, indicating that Miro is also a target in the mitophagy injury induced by the LRRK2 mutation [104,105].

Several studies have suggested a role for LRRK2 in PINK1 and Parkin-mediated mitophagy. A recent study showed that RAB10, a substrate of LRRK2, accumulated on damaged mitochondria in a PINK1-dependent and Parkin-dependent manner. Subsequently, RAB10 was found to bind OPTN to promote its accumulation and subsequently induced mitophagy, indicating that LRRK2 is involved in PINK1-mediated and Parkin-mediated mitophagy via RAB10 [106]. However, a separate study suggested that LRRK2 attenuated PINK1-dependent and Parkin-dependent mitophagic clearance via its kinase activity [107].

### 3.4. DJ-1

Mutations in DJ-1, which is encoded by the *PARK6* gene, cause a rare form of autosomal recessive PD [108]. Upon stress, DJ-1 localizes to mitochondria and acts as a redox sensor/reductase, and its depletion led to mitochondrial deficits and an increase in ROS levels. DJ-1 is regarded as a neuroprotective factor, and mitochondria-localized DJ-1 regulates the clearance of endogenous ROS [109]. DJ-1 deficiency led to an increased level of oxidative stress [110], which is usually associated with mitophagy impairment [111]. The loss of DJ-1 caused increased Parkin recruitment to damaged mitochondria and increased mitophagy, and DJ-1 levels accumulated on mitochondria under oxidative stress conditions dependent on Parkin and PINK1, suggesting a link between DJ-1 and the PINK1/Parkin-mediated pathway [112]. Parkin regulated DJ-1 levels via a signaling cascade implying p53, indicating that p53 and DJ-1 acted acting downstream of parkin [113]. Additionally, a separate study suggested that DJ-1 functioned in parallel with the PINK1/Parkin pathway to maintain mitochondrial function under an oxidative environment [114]. Another study showed mitochondrial defects in DJ-1 knockout flies, which is similar to PINK1 and Parkin mutants. Interestingly, DJ-1 overexpression rescues the phenotype of flies that are deficient for PINK1, but not Parkin. These data also suggest that DJ-1 is critical for mitochondrial function and acts in parallel to or downstream of PINK1 [115].

### 3.5. GBA1

*GBA1* encodes the lysosomal enzyme glucosylceramidase beta/β-glucocerebrosidase, and its heterozygous mutations are among the most common genetic risk factors of PD [116]. GBA1 homozygous mutations cause Gaucher’s disease (GD), which is the most frequent lysosomal storage disorder, and some GD patients and their relatives show parkinsonian manifestations [117]. It has been estimated that GBA1 mutations lead to a 20-fold to 30-fold increased risk of PD, and at least 7–10% of PD patients have a GBA1 mutation [118]. Autophagy defects have been confirmed in iPSC-derived neurons from GBA1-associated PD patients [119], and mitochondrial function was also impaired in GD patients [120]. Additionally, GBA1 deficiencies resulted in α-syn aggregation in PD or GD models and patients [121,122,123], indicating that GBA1 is involved in α-syn pathology and PD pathogenesis, likely by impairing autophagy and mitochondrial function.

In primary neurons from GBA1-knockout mice, autophagy was impaired and mitochondrial function was profoundly compromised with a reduced membrane potential [123]. In GBA^L444P/WT^ knock-in mice, the L444P mutation results in mitochondrial dysfunction by inhibiting mitochondrial priming and autophagy, which are two critical steps for mitophagy. In type II neuronopathic GD, the downregulation of mitophagy resulted in the accumulation of insoluble α-syn deposits [48]. Furthermore, impaired mitophagy and excessive oxidative stress were found in post-mortem brain tissue from PD patients carrying heterozygous GBA mutations, suggesting a link between mitophagy dysfunction and GBA heterozygous mutations [124].

### 3.6. Other PD-Related Proteins

Vacuolar protein sorting-associated protein 35 (Vps35), which is encoded by *PARK17*, causes autosomal-dominant, late-onset PD. Vps35 deficiency or mutation resulted in mitochondrial dysfunction and the loss of dopaminergic neurons [8,125]. Vps35 interacts with Parkin, but not with Pink1; furthermore, its overexpression rescues several Parkin-mutant phenotypes [126].

F-box only protein 7 (Fbxo7), which is encoded by *PARK15*, is a PD-related gene. Mutations of Fbxo7 cause autosomal recessive juvenile atypical PD. Studies showed that Fbxo7 mutation impairs mitophagy, suggesting that Fbxo7 participated in the modulation of mitochondrial homeostasis [127]. Wild-type Fbxo7 can promote mitophagy in response to stress, while mutant Fbxo7 was found to inhibit mitophagy [128]. Another study found that Fbxo7 participates in mitophagy by interacting with PINK1 and Parkin directly, while its PD-related mutations interfered with this process [129]. A PD-related Fbxo7 mutation also recruited Parkin to damaged mitochondria and promoted its aggregation [130].

## 4. Modulation of Mitophagy in PD Treatment

Mitochondrial deficits and autophagy impairment are critical aspects of PD pathogenesis, with impaired mitophagy found in the brains of PD patients and models. Therefore, correcting mitophagy is a promising avenue for the development of efficient treatments for PD. Pharmacological agents that selectively modulate mitophagy are currently lacking, and thus the clinical applicability of this approach remains limited [131]. Although some compounds such as trifluorocarbonylcyanide phenylhydrazone and the combination of antimycin/oligomycin were found to trigger mitophagy, their effects were toxic and non-specific. Therefore, these agents are not suitable for PD treatment [132]. Campanella et al. developed a compound, P62-mediated mitophagy inducer (PMI), that activates endogenous mitophagy without Parkin recruitment or the dissipation of mitochondrial membrane potential. Therefore, PMI has been regarded as a promising chemical candidate [133]. However, whether PMI exerts a therapeutic effect in PD models remains unclear. One recent study suggested that several pathogenic Parkin variants impaired mitophagy, and thus targeting these variants in the design of genotype-specific drugs represents a promising direction [134].

Thus far, several synthetic and natural chemical compounds have been used to trigger mitophagy for treating PD models [10,135]. An interesting study showed that a carrier with a homozygous Parkin mutation resulting in the loss of functional Parkin had not developed PD by her eighth decade of life, indicating the potential existence of a putative mechanism of protection against PD. Further study showed that this carrier had preserved mitochondrial function, and mitophagy could be mediated by mitochondrial receptor Nip3-like protein X (Nix), which is a pathway independent of PINK1/Parkin [136]. This finding suggests that Nix may be a promising target for PINK1/Parkin-related PD treatment, because it could serve as an alternative mediator of mitophagy [137,138].

**Figure 3:**
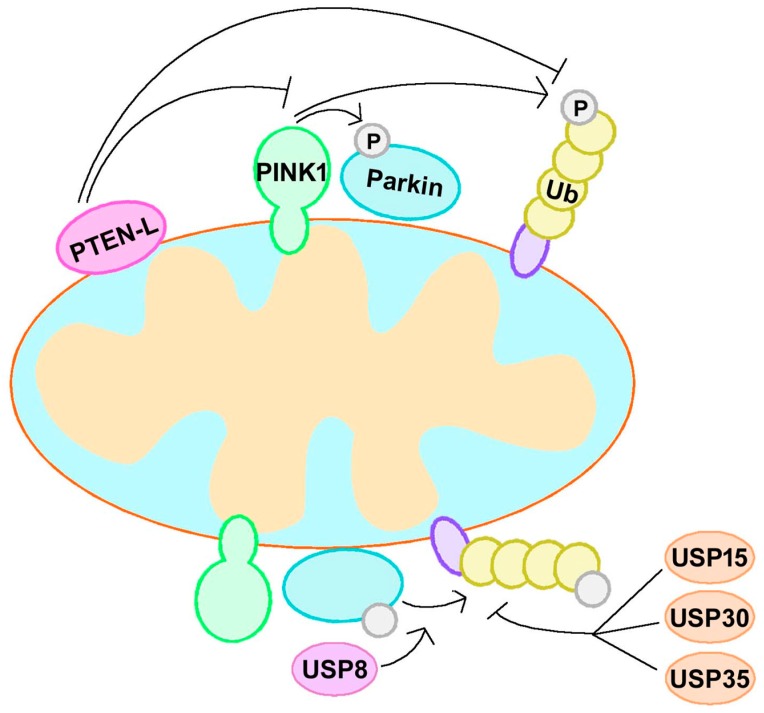
The PINK1–Parkin–Ub feedforward loop. PINK1, Parkin, and Ub form a feedforward mechanism of mitophagy. Regulation of this feedforward loop is a strategy for mitophagy modulation and PD treatment. PTEN-L dephosphorylate p-Ub and suppress mitophagy via blockage of the feedforward mechanism. USP30, USP35, and USP15 can counteract Parkin activity and regulate mitophagy negatively, while USP8 regulate Parkin and mitophagy positively. PTEN-L, phosphate and tension homology deleted on chromsome ten-long; Ub, ubiquitin; USP, ubiquitin specific protease.

The phosphorylation of Parkin and Ub mediated by PINK1 and ubiquitination-mediated Parkin form a feedforward mechanism of mitophagy [52]. Therefore, regulation of the PINK1–Parkin–Ub feedforward loop is a strategy for mitophagy modulation and PD treatment. PTEN-L can dephosphorylate p-Ub and suppress mitophagy via blockage of the feedforward mechanism [63], so the inhibitor of PTEN-L may contribute to mitophagy activation. As mentioned above, USP30, USP35, and USP15 can counteract Parkin activity and regulate mitophagy negatively. The depletion of USP30 enhanced mitochondria degradation in neurons [57]. Similarly, the knockdown of USP35 or USP15 also promoted the mitophagy pathoway [58,61]. However, another DUB, USP8, may regulate Parkin and mitophagy positively, because it removed K6-linked ubiquitin chains from Parkin preferentially to promote the efficient recruitment of Parkin and induction of mitophagy [60]. Although research targeting the PINK1–Parkin–Ub feedforward loop is still limited, it is a valuable direction in PD treatment (Figure 3).

## 5. Conclusions

Many PD-related genes or risk factors are associated with mitophagy defects. There is a strong reciprocal relationship between mitochondria and autophagy, with the impairment of one process usually resulting in damage to the other, and this vicious cycle eventually contributes to the pathogenesis of PD. However, there are some remaining questions. Although the modulation of mitophagy is regarded as a potential approach in PD treatment, whether mitopagy can be specifically targeted is always a question. Although the PINK1–Parkin–Ub feedforward loop is critical for the regulation of mitophagy, its mechanism is still obscure. Despite these, there is a a critical need to understand mitochondrial and lysosomal dysfunction and how their interplay participate in PD pathogenesis, as targeting these crosstalks and restoring mitophagy represent a promising therapeutic approach for PD.

## Figures and Tables

**Table 1:** Overview of Parkinson’s disease (PD)-related genes. Genes related to PD pathogenesis are listed in Table 1. The locus of genes, hereditary properties, and the onset of disease are described. AD, autosomal dominant; AR, autosomal recessive.

Loci	Gene	Protein	Position	Inheritance	Onset
PARK1	*SNCA*	Alpha-synuclein	4q21	AD, sporadic	Early or late
PARK2	*PRKN*	Parkin	6q25–q27	AR, sporadic	Early
PARK3	*Unknown*	Unknown	2p13	AD	Late
PARK5	*UCHL1*	Ubiquitin C-Terminal Hydrolase L1	4p14	AD	Late
PARK6	*PINK1*	PTEN-induced putative kinase 1	1p35–p36	AR	Early
PARK7	*DJ-1*	Protein DJ-1	1p36	AR	Early
PARK8	*LRRK2*	Leucine-rich repeat kinase 2	12q12	AD, sporadic	Early or late
PARK9	*ATP13A2*	ATPase 13A2	1p36	AR	Early
PARK10	*Unknown*	Unknown	1p32	Unknown	Unknown
PARK11	*GIGYF2*	GRB10 interacting GYF protein 2	2q36–q37	AD	Late
PARK12	*Unknown*	Unknown	Xq21–q25	Unknown	Unknown
PARK13	*HTRA2*	Serine peptidase 2	2p13	AD	Late
PARK14	*PLA2G6*	Phospholipase A2 Group VI	22q12–q13	AR	Early
PARK15	*FBX07*	F-Box protein 7	22q12–q13	AR	Early
PARK17	*VPS35*	Vacuolar protein sorting 35	16q11.2	AD	Late
PARK18	*EIF4G1*	Eukaryotic translation initiation factor 4 gamma, 1	3q27.1	AD	Late
PARK19	*DNAJC6*	DNAJ subfamily C member 6	1p31.3	AR	Early
PARK20	*SYNJ1*	Synaptojanin-1	21q22.11	AR	Early
PARK21	*DNAJC13*	DNAJ subfamily C member 13	3q22.1	AD	Early
PARK22	*CHCHD2*	Coiled coil-helix-coiled coil-helix domain 2	7p11.2	AD	Late
PARK23	*VPS13C*	Vacuolar protein sorting 13 homolog C	15q22.2	AR	Early
-	*GBA*	Glucocerebrosidase	1q21	AD	Unknown

**Table 2:** Overview of mitophagy or mitochondrial dynamics deficits in PD transgenic models. Overexpression or deficiency of PD-related genes, including *SNCA, PRKN, PINK1, DJ-1, LRRK2, and GBA*, contributes to mitophagy or defects in mitochondrial dynamics. α-syn, α-synuclein; DA, dopaminergic; GBA, glucocerebrosidase; LRRK2, Leucine-rich repeat kinase 2; PINK1, PTEN-induced putative kinase 1.

Gene	Model	Motor Deficits	Loss of DA Neurons	α-syn Pathology	Mitophagy Defect	Mitochondrial Dynamics Defect
*SNCA*	Wide-type α-syn overexpression	+	+	+	+	+
	A53T α-syn overexpression	+	+	+	+	+
	A30P α-syn overexpression	+	+	+	-	-
*PRKN*	Parkin deficiency	-	-	-	+	+
*PINK1*	PINK1 deficiency	-	-	-	+	+
*DJ-1*	DJ-1 deficiency	+	-	-	+	+
*LRRK2*	G2019S LRRK2 overexpression	+	+	-	+	+
*GBA*	L1444P GBA overexpression	+	unknown	+	+	+
	GBA deficiency	+	unknown	+	+	+
